# Management of failed TFCC repairs

**DOI:** 10.1186/1753-6561-9-S3-A40

**Published:** 2015-05-19

**Authors:** Jason Harvey

**Affiliations:** 1 Orthosport Victoria, Richmond, Victoria, 3121, Australia

## 

Despite appropriate management of distal radius fractures, TFCC tears and DRUJ instability in the acute setting, a subset of patients will have ongoing pain or instability of the DRUJ.

Ongoing instability is thought to eventually lead to pain, loss of motion and degenerative changes due to alterations in normal kinematics of the joint, although there is little in the literature to support this hypothesis.

With an ever increasing emphasis on evidence based medicine, the treatment of asymptomatic instability is therefore difficult to justify as there are no long term natural history studies to document the definitive progression to clinical problems.

The symptomatic patient however presents a different situation entirely. Stability of the DRUJ is conveyed by both static and dynamic influences.

## Static restraints

Bony: a larger radius of curvature sigmoid notch compared to a smaller ulna head, which inherently allows rotation and translation and therefore relative instability.

The volar portion of the sigmoid notch usually has an osteocartilaginous lip to aid in prevention of volar translation of the ulna head.

Ligamentous structures: primarily the volar and dorsal radioulnar ligaments with contributions by the capsule, ulnocarpal ligaments, the interosseous membrane and the TFCC

## Dynamic restraints

The ECU and its subsheath along with the pronator quadratus, which originates just proximal to the joint and envelops the volar aspect of the ulna.

## Assessment

Initial assessment involves history and examination. History includes mechanism of injury, pre-injury symptoms and initial management of the injury and associated injuries as these all can help elucidate the causes of ongoing problems. What are the complaints of the patient? Is it pain, a feeling of instability or frank instability?

On examination the degree of instability, the direction and the exacerbating movements should be evaluated. Assessment in neutral, pronation and supination as well as stress manoeuvres to replicate the instability are useful. It is also important that the contralateral “normal” side is assessed as there is variation in the amount of laxity of the DRUJ between individuals.

The most common finding is subluxation of the ulna dorsally relative to the radius with the forearm in pronation, although this is not universal.

Evaluate for carpal supination and ulnocarpal sag which can accentuate the instability.

## Investigations

### X-ray

Standard PA, lateral and oblique images evaluating both the distal radius for evidence of malunion or sigmoid notch abnormality, the head of the ulna for styloid fractures, ulna head fracture, the joint space for evidence of arthrosis or translation of the ulna relative to the radius or widening of the DRUJ. Contralateral images should be considered if there is any doubt.

### CT Scan

Provides useful information on the morphology of the sigmoid notch in coronal, sagittal and axial planes and is more sensitive for evidence of arthrosis. Scanning the contralateral wrist in the same position may help to define more subtle abnormalities.

It can also be used to define the degree of instability. Several methods have been described to assess this including:

1/ Radioulnar lines

2/ Congruency method

3/ Epicentre method

4/ Radioulnar ratio method

More recently the use of dynamic CT scanning allows real time evaluation of the instability and patients can recreate the instability while being imaged.

## Treatment options

### Non operative

Trial of bracing

Strengthening

Correction of carpal supination/ulnocarpal sag

### Operative

Indications for surgery are pain, loss of motion, weakness or frank instability

Address any bony abnormalities first as any reconstruction of the soft tissues depends upon the integrity of the bone as well. This may include correction of dorsal angulation in the distal radius and consider osteoplastypf the sigmoid notch if it is flattened or altered due to injury.

Once bony issues have been addressed and there is no evidence of arthrosis, soft tissue reconstructive options can be considered. A number of different procedures have been described and were outlined by Kakar et al in 2010.

These include

1/ Creating a tether between the radius and the ulna proximal to the DRUJ using a tendon graft as described by Fulkerson and Watson in 1978

2/ Using an ulnar based flap of extensor retinaculum and advancing it to the distal radius to create a capsulorrhaphy as described by Herbert and Stanley

3/ Creating an ulnocarpal sling or tenodesis as per Hui and Linscheid or Boyes and Bunnell

4/ Reconstruction of the volar and dorsal radioulnar ligaments using a tendon graft as described by Adamsand Berger

## Summary

Pain and instability on the ulna side of the wrist can be a challenging problem to treat. Like most orthopaedic problems a stepwise evaluation of the problem will lead to a better understanding of the issues involved and the potential treatments available.

